# Nitrogen Deficiency Outperforms Water Stress in Triggering Strigolactone‐Dependent Responses in Maize

**DOI:** 10.1111/ppl.70987

**Published:** 2026-07-09

**Authors:** Leonardo Buzzicotti, Claudia Camilletti, Laura Ravazzolo, Markus Wirtz, Rüdiger Hell, Silvia Quaggiotti

**Affiliations:** ^1^ Department of Agronomy, Food, Natural Resources, Animals and Environment University of Padova Legnaro Italy; ^2^ Centre for Organismal Studies Heidelberg University Heidelberg Germany

## Abstract

Strigolactones (SLs) modulate multiple aspects of plant development and stress physiology. This study investigated their role in maize response to abiotic stress by comparing an SL‐biosynthesis mutant (*zmccd8*) with wild‐type (WT) seedlings grown for 4 weeks in vermiculite under nutrient and water limitation. Plant growth, time‐course pigment accumulation, targeted gene expression, and root transcriptomic profiles were analyzed. Our results showed that *zmccd8* plants were largely unable to induce leaf senescence and efficient nutrient remobilization toward younger tissues under nitrogen (N) deficiency, a response previously associated with maize adaptation to low N availability. In parallel, the mutant developed a smaller root system, mainly due to limited adventitious root formation, particularly under N shortage. Root transcriptomic profiling revealed that N deficiency strongly affected WT plants, inducing extensive regulation of pathways involved in nitrogen metabolism and transport, secondary metabolism, ethylene and MAPK signaling, oxidative stress responses, and major transcription factor families. These responses were largely absent in the *zmccd8* mutant, suggesting reduced transcriptional plasticity and compromised capacity to cope with stress‐associated oxidative imbalance. Conversely, despite inducing substantial physiological and molecular responses, water stress elicited only modest SL‐dependent regulation, with limited and heterogeneous changes between genotypes. Overall, our findings demonstrate that in maize, SLs act in a stress‐specific manner, playing a predominant role in acclimatisation to nitrogen deficiency through coordinated regulation of senescence, nutrient remobilization, root architecture, and gene expression, while contributing more marginally to water‐stress acclimatisation. These results provide new insights into SLs' role in shaping maize physiological plasticity under abiotic stress conditions.

## 1 Introduction

Global climate changes intensify the occurrence of abiotic stresses, which severely impair plant nutrient and water uptake, thereby disrupting cellular homeostasis and compromising both growth and metabolism (Barzana et al. 2021). This ultimately reduces plant productivity, particularly in staple crops such as maize ( *Zea mays* L.).

Nitrogen (N) is often the most limiting nutrient for plant growth (Mason et al. 2022), while drought causes greater annual yield losses than all pathogens combined. Acclimatisation to fluctuating N and water availability involves complex physiological and molecular mechanisms that integrate internal and external signals, driving biochemical and developmental adjustments (Schachtman and Shin 2007; Gupta et al. 2020). Understanding the signaling pathways controlling nitrogen use efficiency (NUE) and water use efficiency (WUE) is therefore essential to improve stress physiology knowledge and develop crop varieties adapted to low‐input systems (Fess et al. 2011). Plant hormones play a central role in coordinating plant growth, development, and resource allocation in response to environmental cues. They also regulate acclimatisation to both N deficiency (Kiba et al. 2011) and drought stress (Bailey‐Serres et al. 2019). Among others, SLs have been recognized as integral components of this hormonal network, with substantial evidence supporting their role in plant adaptation to abiotic stress (Saeed et al. 2017).

SLs are carotenoid‐derived terpenoid lactones that regulate shoot and root architecture (Gomez‐Roldan et al. 2008; Umehara et al. 2008), stimulate Orobanchaceae seed germination (Cook et al. 1966), promote AMF hyphal branching (Akiyama et al. 2005), and enhance plant stress tolerance (Kleman and Matusova 2023). In maize, N deficiency strongly induces SL biosynthesis in roots. This response involves changes in polar auxin transport, affecting root elongation (Manoli et al. 2016) as well as adjustments in lateral root (LR) density through both auxin‐dependent and ‐independent pathways (Ravazzolo et al. 2019, 2021). The involvement of SLs in the response to N deficiency has also been reported in rice (Luo et al. 2018), wheat (Sigalas et al. 2024), and tomato (Marro et al. 2022). Moreover, several studies highlight a prominent role for SLs in drought tolerance, mainly due to their interactions with abscisic acid (ABA), as demonstrated in *Arabidopsis* (Ha et al. 2014; Liu et al. 2015). Moreover, exogenous application of the synthetic SL analogue GR24 has been shown to alleviate drought stress in multiple plant species, including maize (Sedaghat et al. 2017, 2020, 2021; Wang et al. 2021; Sattar et al. 2022; Visentin et al. 2020; Min et al. 2019).

The first steps of SL biosynthesis occur in plastids and involve the isomerization of all‐trans‐β‐carotene into 9‐cis‐β‐carotene, catalyzed by the isomerase DWARF27 (D27) (Lin et al. 2009). This is followed by oxidative cleavage of specific double bonds by the non‐heme iron enzymes CAROTENOID CLEAVAGE DIOXYGENASES (CCD7 and CCD8) (Alder et al. 2012). Together, D27, CCD7, and CCD8 form the core pathway that produces the precursor carlactone (CL) (Seto et al. 2014), which is subsequently oxidized into bioactive SL precursors by the cytochrome P450 monooxygenase CYP711A/MAX1 (Abe et al. 2014). Previous studies have demonstrated that transcriptional regulation of *ZmCCD8* is a reliable proxy for predicting SL biosynthesis in maize (Ravazzolo et al. 2019, 2021).

The *zmccd8* maize mutant carries a Dissociation (Ds) transposon insertion in the third exon of *ZmCCD8* (*zmccd8*: *Ds* insertion mutant line in the B73 background, Guan et al. 2012). *zmccd8* plants are unable to synthesize SLs and display reduced stature, narrower stalks, smaller ears, diminished root systems, and a mild branching phenotype (Guan et al. 2012). Preliminary comparisons of *zmccd8* and the parental B73 inbred line under both hydroponic and field conditions confirmed the critical role of SLs in acclimatisation to N deficiency and water stress (Quaggiotti et al. 2024; Ravazzolo et al. 2024). However, while hydroponics allows precise control of N supply and enables clear characterization of the correlations between N availability and SL production, it is not suitable for studying water stress.

Field experiments are more realistic but highly variable due to pedoclimatic factors. To overcome these limitations, we used pot experiments with vermiculite, which mimic soil conditions while allowing precise nutrient and water control. WT and *zmccd8* plants were monitored for 28 days under different nutritional and water regimes. Growth, chlorophyll, and anthocyanin levels were measured at multiple time points, and selected gene expression was analyzed in roots, stems, and leaves. A comprehensive root transcriptome analysis was performed by RNA‐seq. By integrating phenotypic, physiological, and transcriptomic data, this study reveals that SLs modulate maize responses to abiotic stress in a stress‐specific manner, playing a more prominent role under nitrogen deficiency than under water stress.

## 2 Materials and Methods

### 2.1 Plant Material, Growth Conditions and Sampling

Seeds of maize ( *Zea mays* L.) inbred line B73 (wild‐type, WT) and the *zmccd8*: Ds insertional mutant in the same genetic background were surface‐washed and germinated on moist filter paper in the dark at 25°C, as previously described by Quaggiotti et al. (2024). Uniform seedlings were transplanted into pots (7 × 7 × 18 cm) containing 100% vermiculite and divided into four treatments.

Control (CT) plants received complete Hoagland solution three times per week. Nitrogen‐deficient (0N) plants were supplied with the same solution except that 1 mM KNO_3_ was replaced by equimolar KCl. Nutritional stress (NS) plants were irrigated only with Milli‐Q water, while water stress (WS) plants received the complete nutrient solution only at transplanting and were not further irrigated. Plants were grown for 4 weeks under controlled conditions (16/8 h light/dark, 23°C/20°C, 40%–50% RH, 280 μmol m^−2^ s^−1^).

At harvest, fresh weight of leaves, stems, and roots was measured. The primary root was separated from shoot‐borne (adventitious) roots for independent analysis. The data represent 15 biological replicates, each being individual plants grown simultaneously.

### 2.2 Optical Assessment of Leaf Chlorophyll and Anthocyanin Content

Chlorophyll and anthocyanin levels were measured in maize leaves from 14 to 28 days after transplanting (DAT) using a DUALEX SCIENTIFIC+ (Force‐A). Non‐destructive measurements were taken approximately every 2 days at the same time on the mid‐section of each measurable leaf, following Gabriel et al. (2019). The data represent 15 biological replicates.

### 2.3 RNA Extraction and cDNA Synthesis

Total RNA was extracted from 100 mg of tissue collected from the third leaf, stem, and whole root system after 4 weeks of growth. Each biological replicate consisted of pooled tissues from four plants.

RNA was isolated using the Spectrum Plant Total RNA Kit (Sigma) following the manufacturer's instructions. Concentration was measured with a NanoDrop 1000 spectrophotometer (Thermo Scientific), and integrity was verified by agarose gel electrophoresis. First‐strand cDNA was synthesized from 500 ng of total RNA using 1 μL of 10 μM oligo‐dT primers, as previously described (Manoli et al. 2012).

### 2.4 Target Selection for Gene Expression Analysis and qRT‐PCR

The expression of three gene groups was analyzed by qRT‐PCR: (i) genes involved in strigolactone (SL) biosynthesis and transport, (ii) in the nitrogen response and remobilization, and (iii) putatively related to the SL regulatory network and/or drought response (Table S1). Primers were designed using Primer3 (v4.1.0; Rozen and Skaletsky 2000). qRT‐PCR was performed on a StepOne Real‐Time PCR System (Applied Biosystems) using SYBR Green according to the manufacturer's instructions and the protocol of Nonis et al. (2007). Each reaction contained 2.5 ng cDNA and included three technical replicates for each of three biological replicates. Amplification specificity was confirmed by melting curve analysis. Relative expression was calculated using the $2^{-\text{ΔΔCT}}$ method (Livak and Schmittgen 2001), with MEP (Zm00001eb257640) as the reference gene (Manoli et al. 2012).

### 2.5 Statistical Analysis

Statistical analyses were performed in RStudio (version 2025.09.1 + 401; https://cran.rstudio.com) using one‐way ANOVA followed by Tukey's HSD test. Different letters indicate statistically significant differences (*p* < 0.05).

### 2.6 RNA‐Seq Library Construction, Sequencing, and Bioinformatic Analysis

Total RNA integrity from root samples was verified using a Bioanalyzer 2100 (Agilent Technologies). Library preparation and sequencing were performed by HaploX Biotechnology Co. (Hong Kong, China) on an Illumina platform. mRNA was enriched using poly‐T magnetic beads, fragmented, and reverse‐transcribed into cDNA. Strand‐specific libraries were generated by incorporating dUTP during second‐strand synthesis. Libraries were end‐repaired, A‐tailed, adapter‐ligated, size‐selected, PCR‐amplified, purified, and quality‐checked by Qubit, qRT‐PCR, and Bioanalyzer prior to sequencing.

Raw reads were filtered with fastp to remove adapters and low‐quality sequences, and quality metrics (Q20, Q30, GC content) were calculated. Clean paired‐end reads were aligned to the maize reference genome (Zm‐B73‐REFERENCE‐NAM‐5.0) using HISAT2 v2.0.5. Transcript assembly was performed with StringTie v1.3.3b, and gene counts were obtained using featureCounts v1.5.0‐p3. Sequences of novel transcripts are reported in Table S2. Gene expression levels were calculated as FPKM. Differential expression analysis was conducted with DESeq2 (v1.20.0), applying Benjamini–Hochberg correction; genes with adjusted *p* ≤ 0.05 and |log_2_FC| ≥ 1 were considered significant. KEGG and GO enrichment analyses were performed using clusterProfiler, with adjusted *p* < 0.05 as the significance threshold.

## 3 Results

### 3.1 Root and Leaf Development Are Affected in *zmccd8*

**FIGURE 1:**
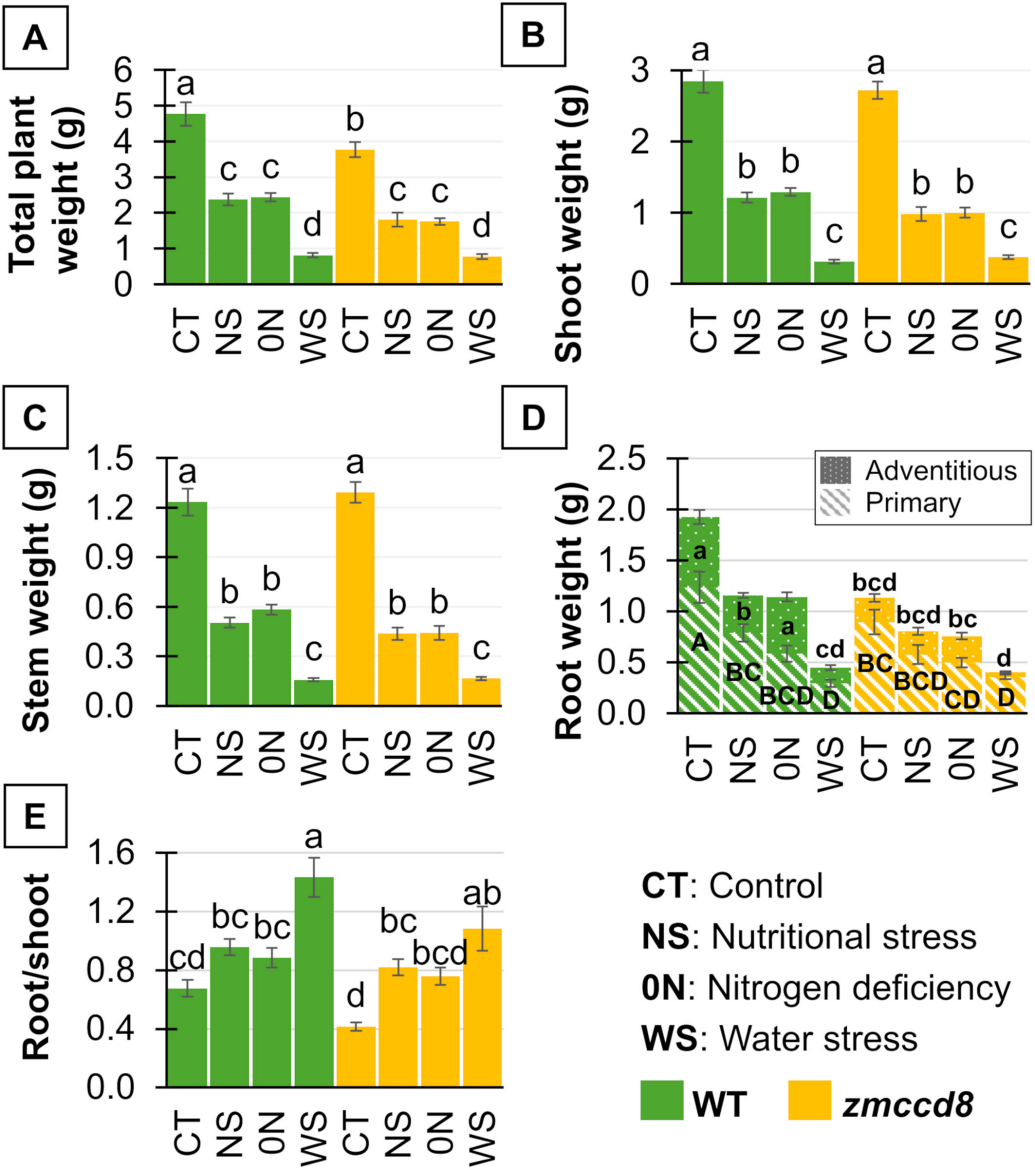
Fresh weight of total plant (A), shoot (B), stem (C), and root (D) tissues in WT (green) and *zmccd8* (yellow) maize seedlings after 4 weeks of growth in vermiculite under different conditions: Control (CT), complete nutrient deprivation (NS), nitrogen deficiency (0N), and water stress (WS). (E) Root‐to‐shoot ratio. Error bars represent mean ± SE (*n* = 15). Statistical analysis was performed using one‐way ANOVA followed by Tukey's HSD test. Different letters indicate statistically significant differences (*p* < 0.05).

Plants were grown as described above and sampled at 28 days after transplanting. *zmccd8* mutants showed a significantly lower overall fresh weight compared to wild‐type (WT) plants under complete nutrient conditions (CT). Moreover, both genotypes exhibited a strong reduction in growth under the three stress conditions, with the highest loss occurring under water stress (WS) and no significant differences between general nutrient stress (NS) and nitrogen‐specific deprivation (0N) (Figure 1A).

The contribution of individual organs was then analyzed. All stress conditions caused reductions in shoot and stem fresh weight, with WS causing the greatest loss and with no significant differences between genotypes (Figure 1B,C). However, root fresh weight in *zmccd8* mutants was significantly lower than in WT under both CT and 0N conditions, indicating that both SLs and nitrogen affect root growth. Water stress also caused a marked decrease in root fresh weight accumulation, with no significant differences between genotypes (Figure 1D).

**FIGURE 2:**
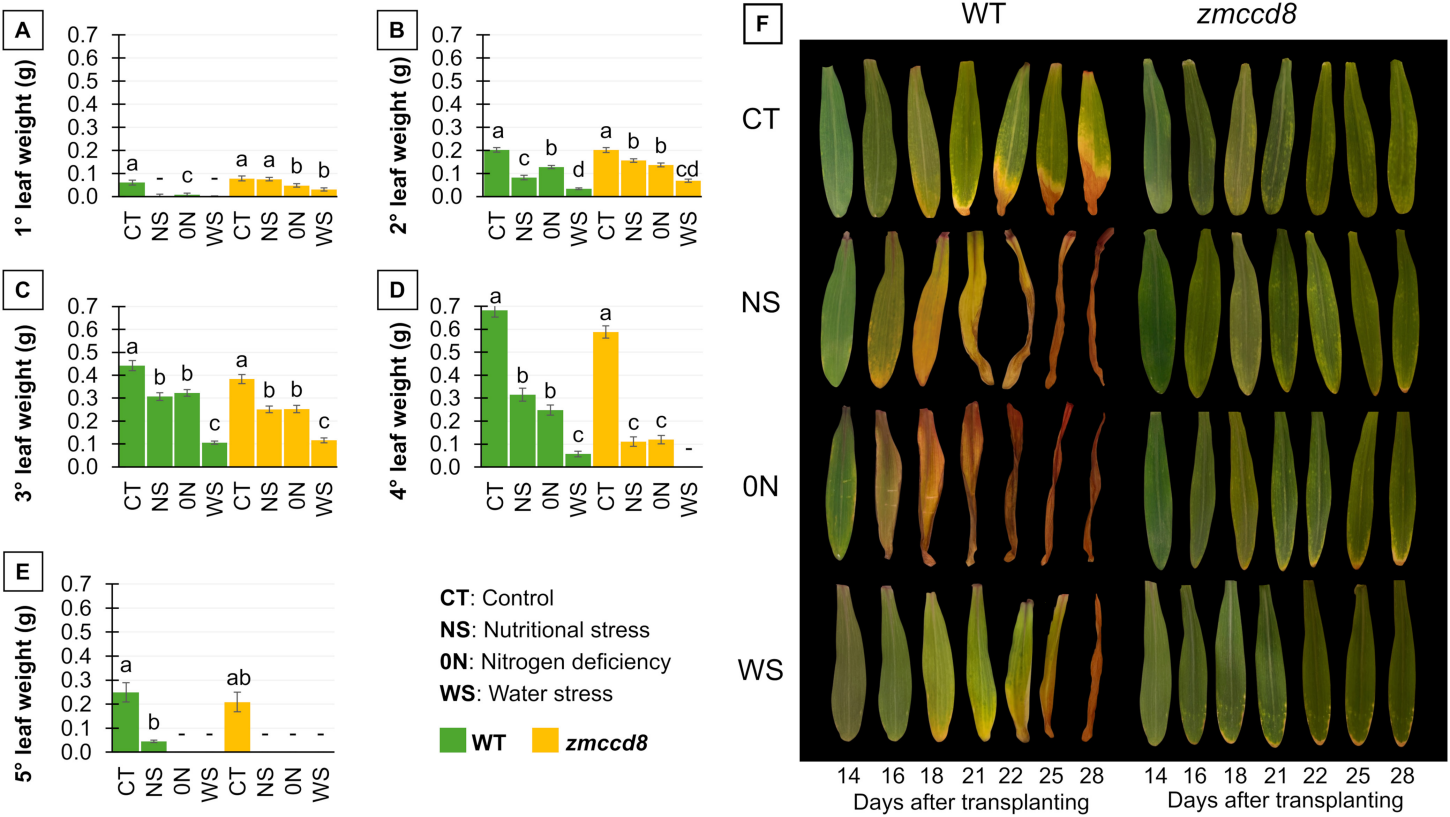
Fresh weight of the first (A), second (B), third (C), fourth (D), and fifth (E) leaves of WT (green) and *zmccd8* (yellow) maize plants after 4 weeks of growth in vermiculite under different conditions: Control (CT), complete nutrient deprivation (NS), nitrogen deficiency (0N), and water stress (WS). Error bars represent mean ± SE (*n* = 15). Statistical analysis was performed using one‐way ANOVA followed by Tukey's HSD test. Different letters indicate statistically significant differences (*p* < 0.05). (F) First leaf phenotype of WT (left) and *zmccd8* (right) seedlings during the final 2 weeks of growth.

Although no differences in total shoot fresh weight were observed between genotypes, individual leaf development was differently affected by nutrient and water deficiencies (Figure 2). In WT, the development of the first leaf was significantly reduced under all stress conditions. In contrast, in *zmccd8*, NS had no significant effect, and WS and 0N caused a milder reduction compared to WT (Figure 2A,F). A similar trend was observed for the second leaf, where WT showed a more pronounced growth reduction compared to *zmccd8* (Figure 2B). The third leaf also showed reduced growth under stress conditions, but no differences were detected between genotypes (Figure 2C). By contrast, the development of the fourth and fifth leaves exhibited a significantly greater reduction in the mutant compared to WT under all stress conditions (Figure 2D,E). These results suggest a genotype‐dependent difference in the capacity for biomass allocation from older to younger leaves in response to stress.

To better assess the relative contribution of primary (PR) and adventitious roots (AR) to the stress response, these root types were analyzed separately (Figure 1D). Under control conditions, WT plants exhibited significantly higher fresh weight for both PR and AR compared with *zmccd8*. In WT, PR fresh weight was strongly reduced by NS, 0N, and WS, whereas in *zmccd8* it was significantly affected only by WS. Moreover, AR fresh weight was significantly lower in *zmccd8* mutants compared to WT under 0N conditions. This pattern is further illustrated in Figure S1F, which shows the proportion of AR relative to total root fresh weight. Under WS, the difference in AR fresh weight between genotypes was not significant; however, the proportion of AR relative to total root was higher in WT than in the mutant.

### 3.2 WT and *zmccd8* Display Distinct Leaf Chlorophyll Content Profiles

Leaf chlorophyll (Chl) content, as a proxy for resource allocation (Onoda et al. 2017), was measured every 2–3 days from 14 to 28 days after transplanting in all four leaves and under the three tested nutritional regimes for both genotypes.

**FIGURE 3:**
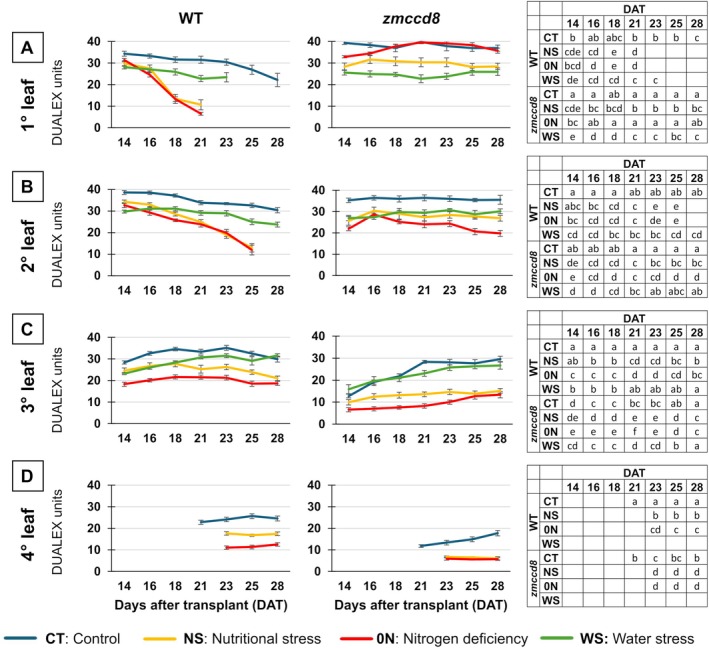
Chlorophyll levels quantified using the optical sensor DUALEX SCIENTIFIC+ (Force‐A) in the first (A), second (B), third (C), and fourth (D) leaves of WT (left panels) and *zmccd8* (right panels) seedlings. Measurements started 14 days after transplanting. Plants were grown in vermiculite under different conditions: Control (CT), complete nutrient deprivation (NS), nitrogen deficiency (0N), and water stress (WS). Error bars represent mean ± SE (*n* = 15). Statistical analysis was performed using one‐way ANOVA followed by Tukey's HSD test. Different letters indicate statistically significant differences (*p* < 0.05).

Chl content of the first fully developed leaf showed a slightly decreasing trend over the course of the experiment in WT plants. This decline was more pronounced under NS and 0N conditions, with consistently and significantly lower values compared to the CT condition. Furthermore, from Day 21 onward, in these conditions, Chl was no longer detectable, likely due to the onset of senescence (Figure 3A). A similar but less pronounced trend was observed under WS. In contrast, the first leaf of *zmccd8* plants maintained a more stable Chl content throughout the experiment across all nutritional regimes. Only minor differences were observed between CT and 0N, mainly at Days 14 and 16. Under WS and NS treatments, *zmccd8* plants had a slightly lower Chl content at all measured time points (Figure 3A).

The second leaf showed a similar pattern. In WT, Chl content declined over time, with NS and 0N treatments having significantly lower values than CT and WS, though no significant differences were observed between NS and 0N themselves. As for the first leaf, senescence induced by NS and 0N was also apparent, albeit with a delayed onset. In *zmccd8* plants, the Chl content in the same leaf remained relatively constant over time, although stress conditions caused a significant reduction compared to CT at all time points (Figure 3B).

In WT plants, the third leaf maintained a more stable Chl content over time, although plants under NS and 0N conditions exhibited significantly lower values than CT and WS. Notably, no signs of senescence were observed in this leaf up to Day 28 (Figure 3C). In *zmccd8* plants, the third leaf under CT and WS conditions showed an increasing trend in Chl accumulation, although overall values were generally lower than those in WT plants. In contrast, both NS and 0N treatments induced a clear reduction in Chl content at nearly all time points (Figure 3C).

The fourth leaf showed higher Chl content in WT compared to *zmccd8*, regardless of the nutritional condition. However, the effects of nutrient stress were evident in both genotypes (Figure 3D). Data for the WS condition are not available, as the fourth leaf had not yet developed in these plants after 28 days (Figure S1D).

Overall, these results suggest that the two genotypes adopt different strategies in response to nutritional stress. During NS and 0N, WT plants appear to remobilize resources from older to younger leaves, leading to stress‐induced senescence primarily in the oldest leaves. In contrast, *zmccd8* plants show a reduced capacity for such remobilization, with younger leaves being more affected under stress conditions. Under WS, differences between the two genotypes were minimal.

### 3.3 WT and *zmccd8* Display Distinct Leaf Anthocyanin Content Profiles

**FIGURE 4:**
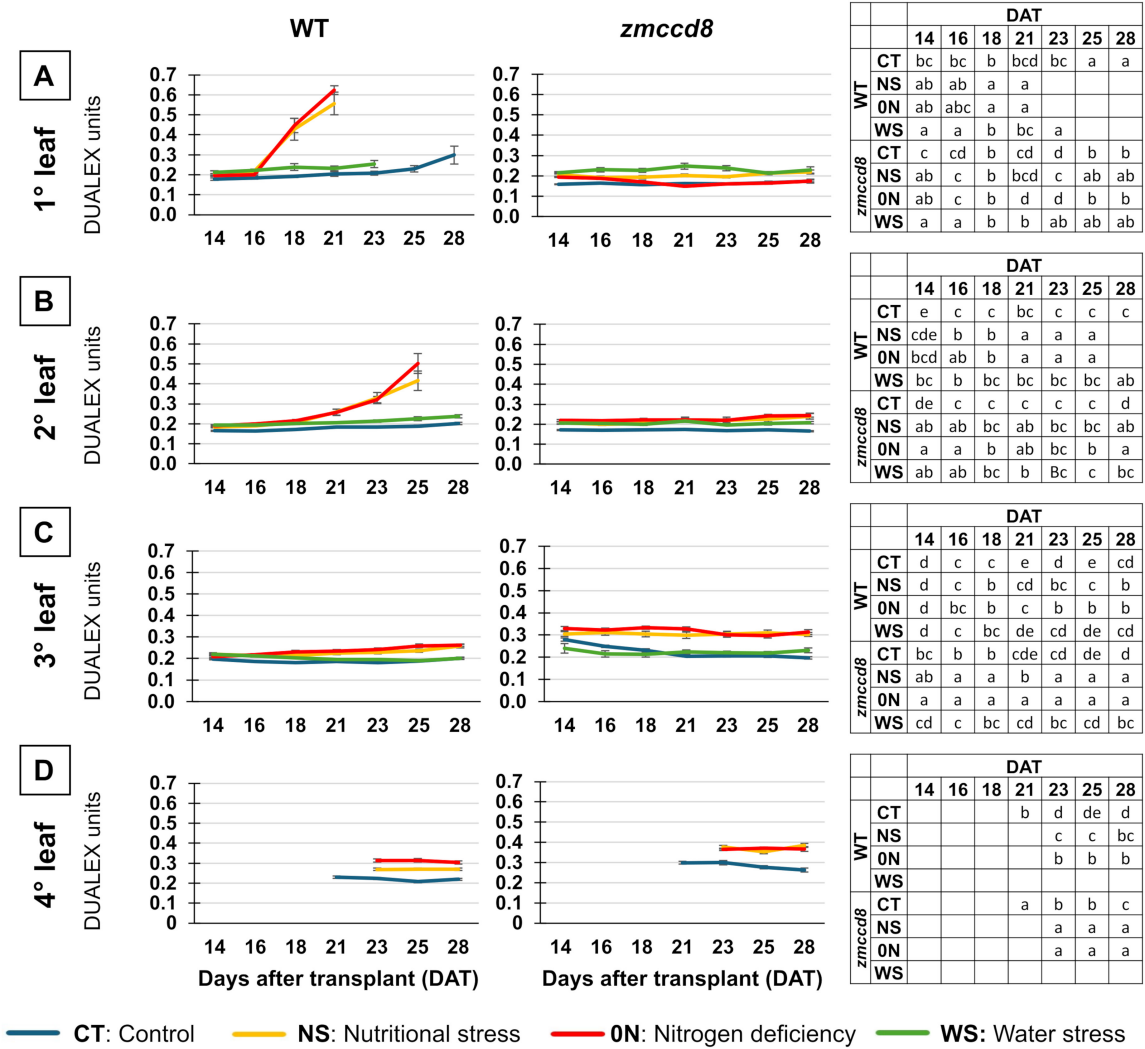
Anthocyanin levels quantified using the optical sensor DUALEX SCIENTIFIC+ (Force‐A) in the first (A), second (B), third (C), and fourth (D) leaves of WT (left panels) and *zmccd8* (right panels) seedlings. Measurements started 14 days after transplanting. Plants were grown in vermiculite under different conditions: Control (CT), complete nutrient deprivation (NS), nitrogen deficiency (0N), and water stress (WS). Error bars represent mean ± SE (*n* = 15). Statistical analysis was performed using one‐way ANOVA followed by Tukey's HSD test. Different letters indicate statistically significant differences (*p* < 0.05).

Anthocyanin accumulation is a common response in plants experiencing N deficiency, acting as a protective mechanism against various stress factors (Ravazzolo et al. 2024). WT consistently exhibited significantly higher levels of these pigments in response to NS and 0N, with a clear increase over time as the treatments progressed. This trend was most evident in the older leaves (first and second), but also appreciable in the third and fourth leaves. In the fourth leaf, the effect of the 0N treatment was even stronger than that of NS (Figure 4A–D).

In *zmccd8*, no time‐dependent increase in anthocyanin accumulation was observed under stress conditions. However, NS and 0N treatments induced slight but generally significant increases in anthocyanin content at all measured time points, with the highest accumulation observed in the third and fourth leaves (Figure 4A–D). Water stress did not appear to influence anthocyanin levels, as their content remained almost constant and similar to the control.

Overall, these results indicate that WT plants markedly increase anthocyanin production in response to both general and nitrogen‐specific nutritional deficiencies, particularly in older leaves and in a time‐dependent manner. In contrast, *zmccd8* plants showed only modest increases in anthocyanin content, with no clear temporal pattern.

### 3.4 Gene Expression Responses to Nutrient and Water Availability

**FIGURE 5:**
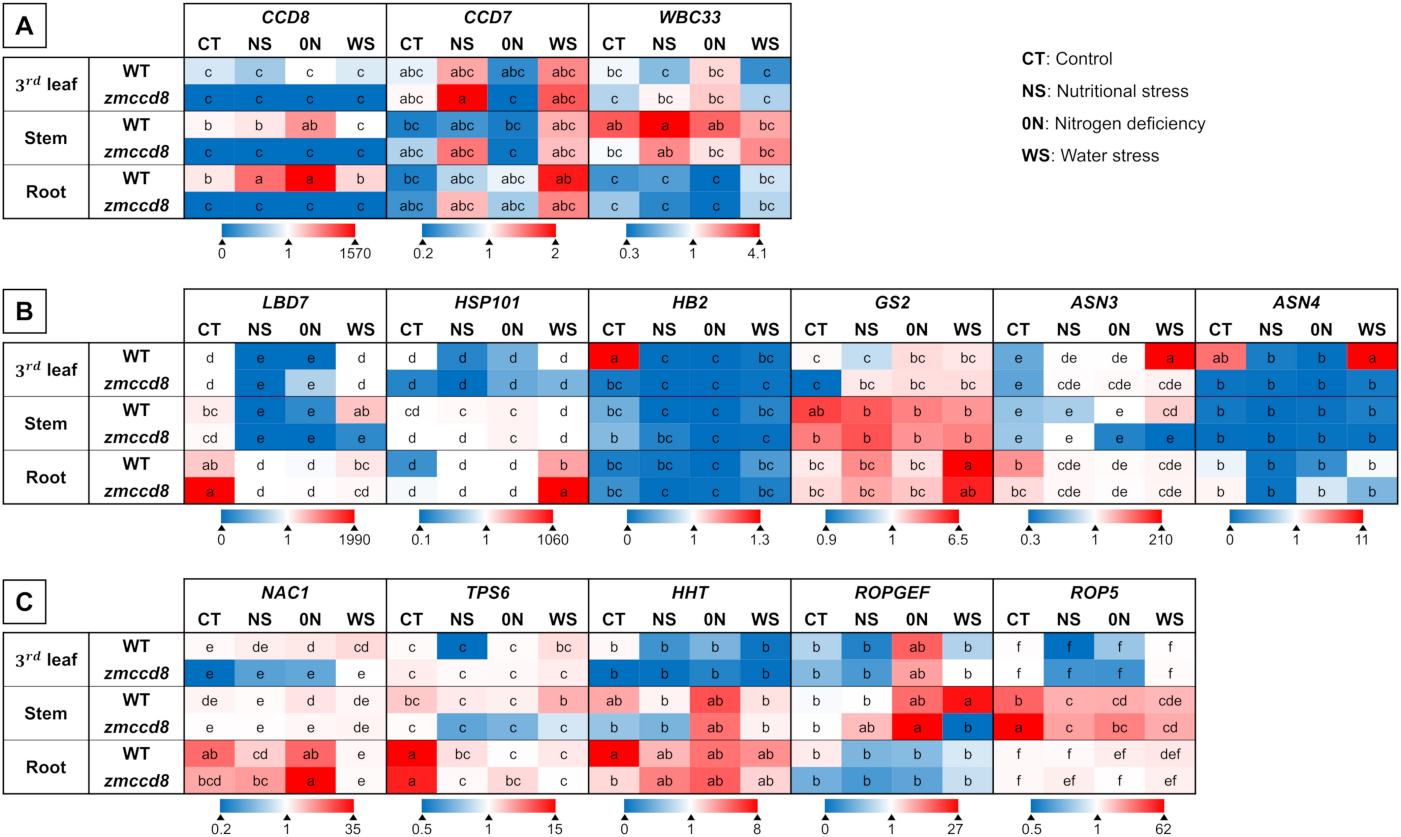
Relative expression levels of genes involved in (A) SL biosynthesis and transport, (B) nitrogen response and remobilization, and (C) putatively related to the SL regulatory network and/or water stress responses in third leaf, stem, and root samples from WT and *zmccd8* seedlings after 4 weeks of growth in vermiculite under different conditions: Control (CT), complete nutrient deprivation (NS), nitrogen deficiency (0N), and water stress (WS). Color intensity of the squares reflects changes in gene expression levels (*n* = 3 biological replicates). Statistical analysis was performed using one‐way ANOVA followed by Tukey's HSD test. Different letters indicate statistically significant differences (*p* < 0.05).

To better characterize the role of strigolactones (SLs) in maize stress responses, the transcriptional profiles of several previously identified genes were analyzed by qRT‐PCR in the third leaf, stem, and root of both genotypes (see Table S1 for gene list). The third leaf was selected as it did not show visible signs of senescence, thus ensuring high‐quality RNA samples and better reflecting the regulatory situation before the onset of the senescence program. Genes were chosen based on their putative involvement in the SL pathway (Figure 5A), nitrogen sensing (Figure 5B), and water response (Figure 5C).

The expression of three SL‐related genes (*CCD8*, *CCD7*, and *WBC33*; Table S1) was analyzed. In WT plants, *CCD8* was slightly upregulated under 0 N in all tissues, with significant differences only in roots, consistent with previous reports (Ravazzolo et al. 2019, 2021), whereas WS had no effect. As expected, *CCD8* transcripts were undetectable in the *zmccd8* mutant (Figure 5A). *CCD7* and *WBC33* were weakly expressed and showed no significant differences among treatments or genotypes (Figure 5A).

Based on previous results, *LBD7*, *HSP101*, *HB2*, *GS2*, *ASN3*, and *ASN4* were selected as nitrogen‐related genes (Table S1). *LBD7* was consistently downregulated under N starvation almost in all tissues and genotypes, especially under nutrient starvation, in agreement with Trevisan et al. (2015); under WS, its stem expression was lower in mutants than in WT (Figure 5B). *HSP101* was induced by 0N in stems and strongly upregulated by WS in roots of both genotypes, with no genotype‐dependent differences, suggesting SL‐independent regulation. *HB2* was significantly more expressed in WT leaf than in *zmccd8* in CT and it was downregulated by stress conditions in WT, whereas *GS2* remained unchanged. *ASN3* was induced by WS in the third leaf and stem of WT but not *zmccd8*, while it was downregulated in roots of WT under all stresses. *ASN4* showed very low expression overall, except in the third leaf of WT under CT and WS (Figure 5B).

Additional genes were selected based on their putative roles in maize water stress responses and SL signaling (Table S1). *NAC1* expression was lower in the third leaf of *zmccd8* than WT under 0N and WS and induced by WS only in WT, with no differences in other tissues (Figure 5C). *TPS6* was downregulated by all stresses in roots, similarly in both genotypes. *HHT*, *ROPGEF*, and *ROP5* showed comparable expression between genotypes, except for reduced *ROPGEF* levels in mutant stems (Figure 5C).

Taken together, the data suggest modest and partly unexpected genotype‐dependent variation at the transcriptional level. This observation underscores the importance of employing untargeted approaches to uncover broader regulatory mechanisms and patterns.

### 3.5 RNAseq Analysis Revealed Genotype‐Specific Differences in the Response to Nitrogen and Water Starvation in Roots

The diverging expression patterns of the selected genes prompted us to adopt a broader untargeted approach to investigate molecular changes occurring in response to different stresses in the two genotypes. To validate previous results, we verified and confirmed the correspondence between the expression profiles reported in Figure 5 and those from the RNA‐seq data. As relevant and specific effects of NS were not observed, it was excluded from these analyses, which were focused on root tissues under CT, 0N, and WS conditions. Roots were chosen because of their crucial role in nitrogen and water uptake and because they showed the most significant differences in fresh weight accumulation between genotypes. RNA sequencing generated between 46.6 and 57.7 million raw reads per root sample. The number of clean reads ranged from 43.5 to 53.7 million, with mapping rates between 96.1% and 98.6%. GC content varied from 49.7% to 54.1%. Q20 and Q30 values were above 99.1% and 96.4%, respectively (Table S3), indicating sequencing data suitable for downstream transcriptomic analysis.

**FIGURE 6:**
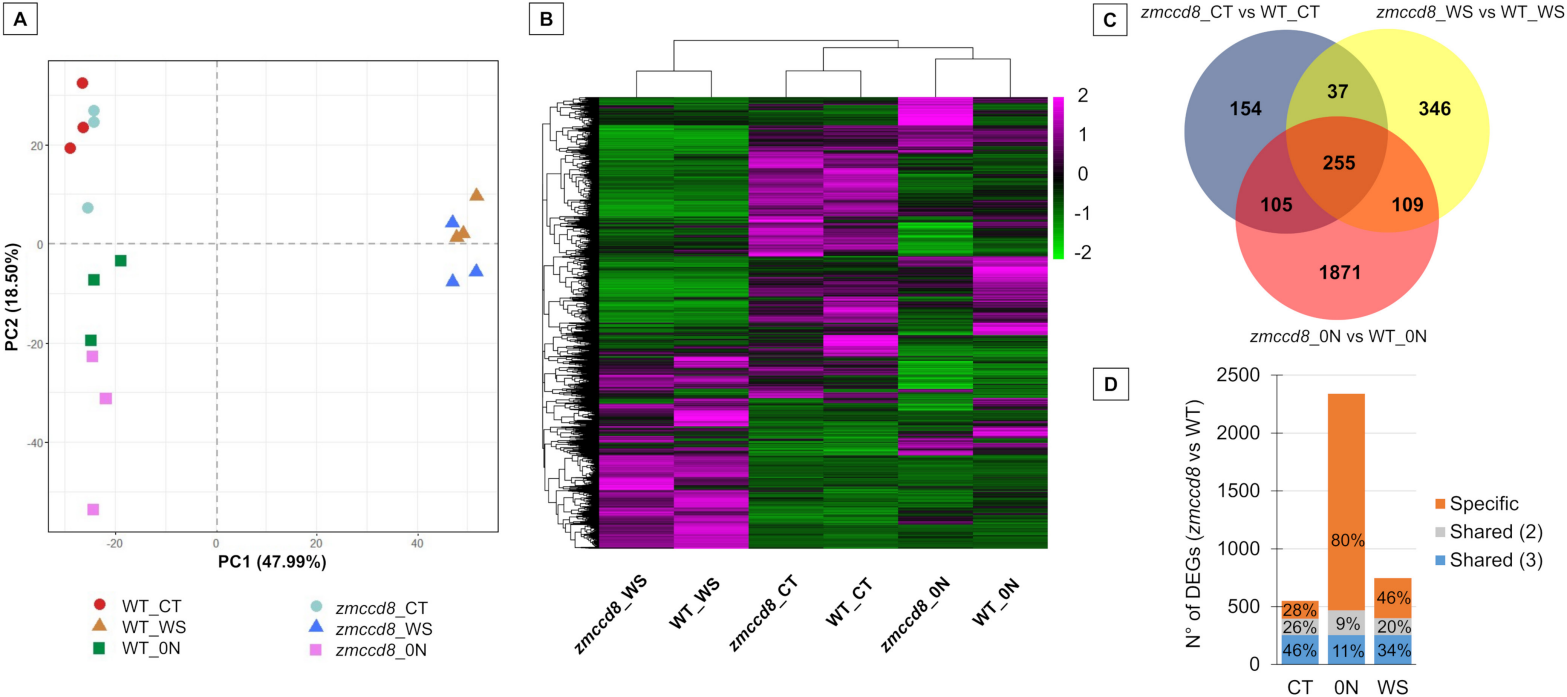
(A) Principal component analysis of gene expression values (FPKM) showing the global transcriptional variance across all samples. (B) Heatmap of differentially expressed genes between the two genotypes displaying the clustering of samples based on $\log_{2}\left(\text{FPKM}+1\right)$ values under CT, 0N, and WS conditions. (C) Venn diagram representing the distribution of differentially expressed genes (DEGs) between the two genotypes across the three conditions. (D) Histogram classifying DEGs as condition‐specific, shared between two conditions, or common to all three treatments.

Principal Component Analysis (PCA) was performed on WT and *zmccd8* samples across the three conditions. The first and second principal components explained 47.99% and 18.50% of the variance, respectively. The analysis revealed that WS (triangles) caused the largest separation from control samples (dots), with data shifting primarily along the first component. In contrast, differences due to 0N (squares) were milder but more genotype‐specific, as WT and *zmccd8* samples formed two clearly separated groups (Figure 6A).

Additional evidence consistent with the PCA results was obtained from the expression heatmap of differentially expressed genes (DEGs) (Figure 6B). WS induced the largest differences in gene expression compared to control, with most DEGs showing almost opposite patterns between stressed and control conditions, but with the two genotypes displaying similar responses. By contrast, under 0N, the overall response was milder, but marked differences emerged between genotypes, with distinct groups of genes showing opposite expression patterns in WT and *zmccd8* (Figure 6B). These findings support the previously hypothesized role of SLs in regulating the overall maize response to nitrogen deficiency and highlight the specificity of their involvement in adaptation to N starvation rather than in water stress tolerance.

Venn diagrams of DEGs further illustrate genotype‐specific responses under the three conditions (Figure 6C). Under control (blue circle), 551 DEGs were detected, of which 154 (28%) were specific for the CT condition (Figure 6D), while the remaining overlapped with those observed in WS, 0N, or both (37, 105, and 255 DEGs, respectively). Under WS (yellow circle), 747 DEGs were identified, 346 (46%) being specific to WS (Figure 6D), 364 overlapping with those detected in the case of nitrogen response, and 292 being already differentially expressed in the CT conditions. Under 0N (red circle), 2340 DEGs were detected, approximately three times the number observed under WS. Of these, 1871 (80%) were specific to the response to 0N (Figure 6D), more than fivefold higher than those observed for the WS response. The remaining DEGs overlapped with those already detected in CT conditions (360) or in the WS response (364). Among genes shared between responses to 0N and to WS, some were also in common with CT (255), while others (109) may represent general stress‐response genes in maize roots (Figure 6C). These results confirm that the two genotypes exhibit distinct transcriptional responses to both stresses, with much stronger divergence between them under 0N than WS.

### 3.6 Functional Enrichment Analysis

**FIGURE 7:**
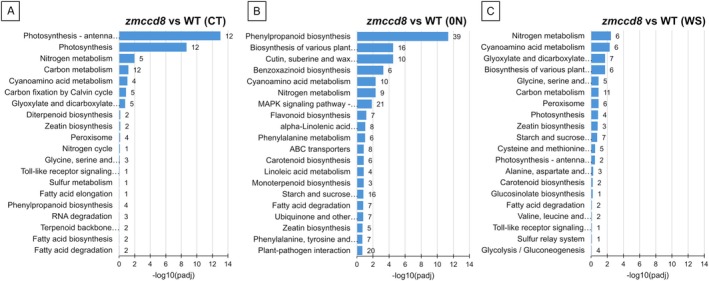
Top 20 enriched KEGG pathways and their significance levels, expressed as –log_10_(padj), under the three experimental conditions: Control (A), nitrogen deficiency (B), and water stress (C). Values next to each bar indicate the number of genes annotated to the corresponding KEGG pathway.

To explore the functional roles of DEGs (Table S4) under the three conditions, KEGG (Figure 7; Table S5) and GO (Figure S2; Table S7) enrichment analyses were performed. As the results were largely concordant, we focused on KEGG. Enrichment results comparing the two genotypes across conditions are described below. Under CT, the most significantly enriched pathways were *photosynthesis, photosynthesis–antenna proteins*, *nitrogen* and *carbon metabolism*, all of which showed lower expression levels in *zmccd8* compared to WT (Figure 7A). Under WS, several pathways were significantly enriched; nevertheless, genes included in each pathway did not follow a consistent expression pattern, with some showing higher expression in the WT and others in the *zmccd8*. These pathways included *nitrogen metabolism*, *cyanoamino acid metabolism*, *glyoxylate and dicarboxylate metabolism*, and *biosynthesis of plant secondary metabolites* (Figure 7C). Within the latter, two *ZmNAS* genes (*NAS6* and *NAS2*) were more highly expressed in the WT genotype, whereas one *ZmNAS* (*NAS5*) and one gene involved in mugineic acid biosynthesis showed higher expression in *zmccd8* (Table S5). Interestingly, the *starch and sucrose metabolism* pathway, although not significantly enriched, comprised several genes all downregulated in *zmccd8* compared with WT (Table 1A).

**TABLE 1:** (A) Genes involved in starch and sucrose metabolism that were differentially expressed between the two genotypes under water stress. (B) Differential expression of maize nitrogen transporter genes between *zmccd8* and WT under the three experimental conditions.

A			
Description	Name	Gene ID	WS
			Log_2_FC
Starch and sucrose metabolism	*ZmDHR2*	Zm00001eb138340	−6.3
	*ZmBGLU15*	Zm00001eb334460	−1.9
	*ZmTRPP1*	Zm00001eb042260	−1.5
	*ZmTPS10*	Zm00001eb192680	−1.1
	*ZmBT2*	Zm00001eb176800	−1.1
	*ZmTPS6*	Zm00001eb178890	−1.0
	*ZmCEL19*	Zm00001eb350640	−1.0

B					
Description	Name	Gene ID	0N	WS	CT
			Log_2_FC	Log_2_FC	Log_2_FC
Nitrate Transporter 1/Peptide Transporter Family	*ZmNPF1.1*	Zm00001eb152400	ns	2.5	ns
	*ZmNPF2.1*	Zm00001eb033080	−1.3	ns	ns
	*ZmNPF3.1*	Zm00001eb380020	−1.3	−1.1	ns
	*ZmNPF3.2*	Zm00001eb287980	ns	−1.0	ns
	*ZmNPF4.2*	Zm00001eb082170	−1.0	ns	ns
	*ZmNPF5.1*	Zm00001eb003170	−1.2	ns	ns
	*ZmNPF5.6*	Zm00001eb211600	−1.6	−1.8	−1.1
	*ZmNPF6.2*	Zm00001eb162570	ns	−2.5	ns
	*ZmNPF6.6*	Zm00001eb023600	−1.8	ns	ns
	*ZmNPF6.8*	Zm00001eb245250	ns	−1.5	ns
	*ZmNPF7.10*	Zm00001eb251130	−3.3	ns	ns
	*ZmNPF8.1*	Zm00001eb335710	−0.9	ns	ns
	*ZmNPF8.2*	Zm00001eb144350	ns	2.4	1.9
	*ZmNPF8.16*	Zm00001eb025880	−1.1	ns	ns
High‐affinity nitrate transporter	*ZmNRT2.5*	Zm00001eb361110	ns	1.2	ns
Ammonium transporter	*ZmAMT2.1*	Zm00001eb289990	−0.9	ns	ns
	*ZmAMT3.1*	Zm00001eb366590	−1.8	ns	ns
	*ZmAMT3.2*	Zm00001eb063910	−1.1	ns	ns

The 0N condition showed the most significantly enriched pathways and the largest genotype differences (Figure 7B; Table S5). The *phenylpropanoid biosynthesis* pathway showed reduced transcriptional activity in the mutant, with six genes encoding phenylalanine ammonia‐lyase (PAL) exhibiting lower expression compared to WT. The *nitrogen metabolism* pathway was enriched, with two nitrate reductase genes and one nitrite reductase gene expressed lower in the mutant. *Carotenoid biosynthesis*, including ABA and SL‐related genes, was enriched in WT, as was the *ABC transporter group*: two ABCB and five ABCG genes were higher in WT, while only one ABCG gene was higher in *zmccd8*. The *MAPK signaling* pathway was enriched under nitrogen deficiency, encompassing transcripts involved in MAPK, ABA, ROS, and Ca^2+^ signaling, key components of plant stress responses (Suzuki et al. 2013; Dubiella et al. 2013). Most of these genes exhibited higher expression levels in the WT than in the *zmccd8* mutant. Other pathways enriched under nitrogen deficiency included *cutin*, *suberin*, and *wax biosynthesis*, *secondary metabolite biosynthesis*, *cyanoamino acid metabolism*, and *benzoxazinoid biosynthesis* (Figure 7B), with most associated genes expressed showing lower expression in *zmccd8* than in WT. To deepen the putative involvement of SLs on nitrogen uptake, transcripts encoding nitrate or ammonium transporters (Jia et al. 2023) were searched among the DEGs (Table 1B). Nine genes encoding *NITRATE TRANSPORTER 1/PEPTIDE TRANSPORTER FAMILY (NPF)* genes and three encoding ammonium transporters (*AMT*) were significantly more expressed in WT in response to nitrogen deprivation.

**FIGURE 8:**
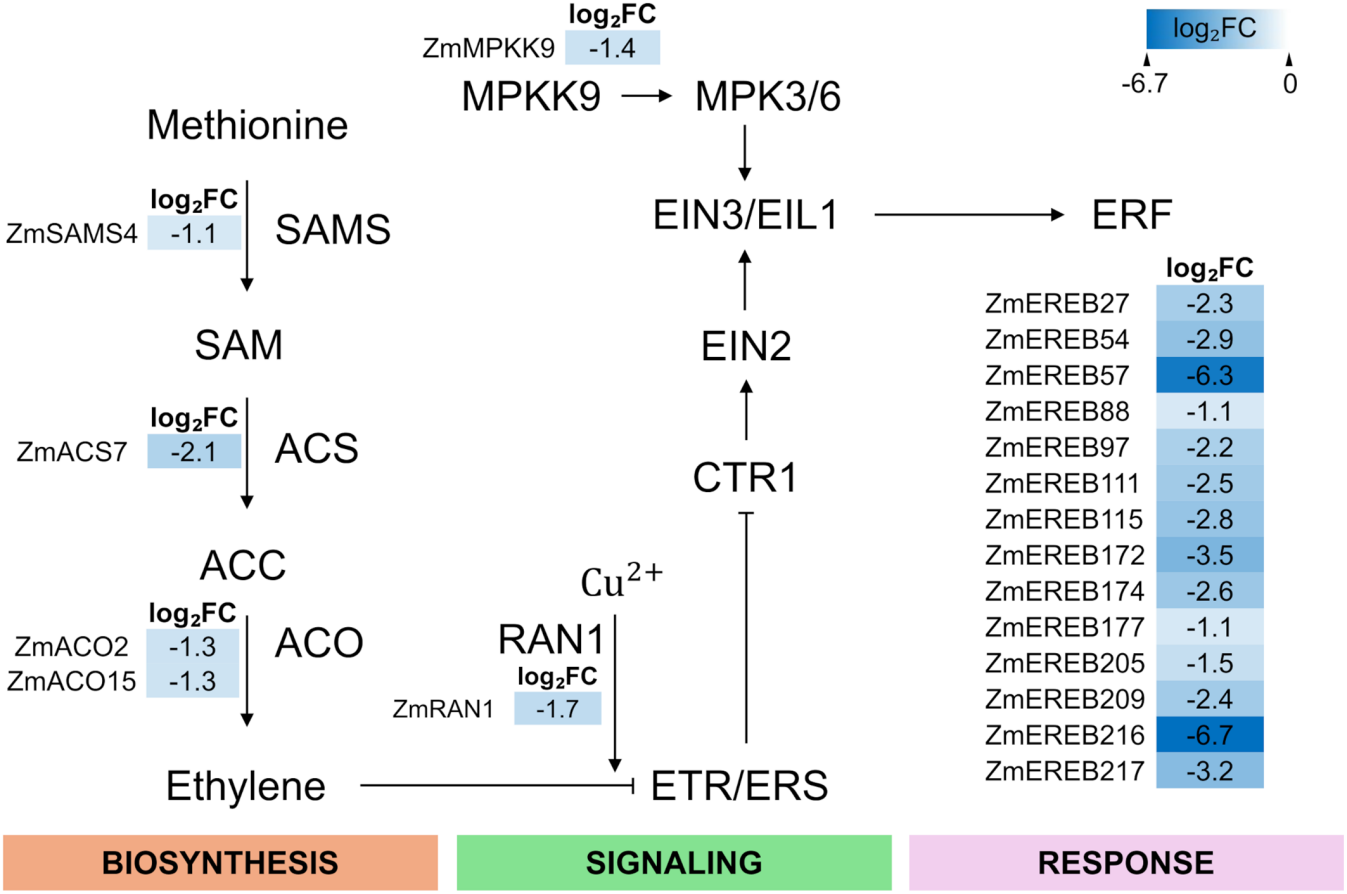
Pathways of ethylene biosynthesis, signaling, and response in maize, with associated differentially expressed genes between the two genotypes under nitrogen deficiency conditions.

In addition, several genes involved in ethylene (ET) biosynthesis, including *SAMS*, *ACS*, and *ACO* (Aragón‐Raygoza and Strable 2025), were expressed at lower levels in the mutant than the WT in response to N deficiency. The same trend was observed for *RAN1*, a copper transporter required for ET receptor activity (Binder et al. 2010), and for *MPK9*, a MAP kinase known to promote ET signaling in *Arabidopsis* (Yoo et al. 2008) (Figure 8; Table S4).

Finally, transcription factors and coregulators were analyzed using GRASSIUS 2.0 (Gray et al. 2024). Under 0N, 165 genes were differentially expressed (|log_2_FC| ≥ 1, padj ≤ 0.05), with 157 higher in WT and nine in *zmccd8*, mainly from MYB (30), AP2/ERF (24), NAC (14), WRKY (11), and bHLH (11) families. Under WS and control conditions, 36 and 25 TF genes were differentially expressed, respectively, with a roughly balanced distribution between genotypes (Table S6).

## 4 Discussion

Maize ( *Zea mays* L.) is a major cereal crop and a key contributor to global food security (FAO 2023). However, the increasing frequency of climate change‐related abiotic stresses severely affects its growth and yield (Zhang, Wei, et al. 2022; Zhang, Zhang, et al. 2022; Kim and Lee 2023). Understanding the mechanisms underlying plant acclimatisation to environmental stress is therefore essential to mitigate climate impacts on crop productivity. In this context, plant hormones play a pivotal role by integrating environmental cues and downstream adaptive physiological processes (Verma et al. 2016; Waadt et al. 2022). Among them, SLs have emerged as a novel class of phytohormones modulating plant development and responses to abiotic stresses, including drought, salinity, and nutrient limitation (Marzec and Muszynska 2015; Mansoor et al. 2024). The maize *zmccd8*:Ds mutant, which is impaired in SL biosynthesis, exhibits altered responses to nitrogen deficiency under hydroponic conditions (Quaggiotti et al. 2024) and to water stress under field conditions (Ravazzolo et al. 2024). In the present study, this mutant and its corresponding wild‐type (WT) were grown in vermiculite, a substrate that more closely mimics natural soil conditions than hydroponics while still allowing precise control of environmental parameters. Plants were subjected to nutrients (NS), nitrogen (0N), and water (WS) deprivation respectively.

In WT seedlings, NS and 0N treatments reduced fresh weight (Figure 2A,B) and chlorophyll content (Figure 3A,B), and increased anthocyanin accumulation (Figure 4A,B) in older leaves. These changes reflect nitrogen deficiency‐induced senescence, which promotes N remobilization to younger tissues and enhances NUE (Sakuraba 2022), as previously reported in maize (Xing et al. 2024). This process appeared to be suppressed in the *zmccd8* mutant, resulting in lower fresh weight (Figure 2A,B) and chlorophyll content (Figure 3C,D) in the younger leaves. Under WS, senescence was also observed in the WT but not in the *zmccd8*, confirming the role of this class of phytohormones in regulating senescence in response to multiple stresses in maize (Hu et al. 2023). These findings indicate that SL biosynthesis is required for efficient nutrient remobilization to younger sinks, particularly under nitrogen deficiency. Recently, Zhong et al. (2025) demonstrated that loss of *ZmCCD8* function reduced kernel growth and sucrose and amino acid accumulation in kernels, supporting the involvement of SLs in nutrient remobilization.

Roots are key for plant adaptation to nutrient and water limitations (Holz et al. 2024), as their architecture can be modulated in response to environmental conditions (López‐Bucio et al. 2003; Keerthi et al. 2025). In maize seedlings, the primary root (PR) dominates early water and nutrient uptake. As development proceeds, shoot‐borne (adventitious) roots emerge and become the main organs for absorption (Hochholdinger 2004; Hochholdinger and Tuberosa 2009; Viana et al. 2022). In our study, *zmccd8* plants showed a less developed root system under control conditions and produced fewer adventitious roots (AR) than the WT (Figure 1D), both under control and nitrogen‐deficient conditions. These results support the role of SLs in regulating maize root architecture (Guan et al. 2012; Manoli et al. 2016; Ravazzolo et al. 2021) and align with rice studies, where SL‐deficient mutants show reduced crown roots (Arite et al. 2012). Under WS, root fresh weight was drastically reduced, and genotypic differences, though still apparent, were no longer statistically significant. The proportion of adventitious roots was higher in WT than in the mutant, suggesting SLs may promote AR development even under water stress. To investigate molecular responses, the expression of genes related to SL biosynthesis/signaling (Figure 5A), nitrogen perception (Figure 5B), and water stress adaptation (Figure 5C) was measured in the third leaf, stem, and roots (Table S1). The two genotypes exhibited distinct gene expression patterns depending on the tissue type and the applied stress condition. *CCD8* was confirmed as a marker of the root response to nitrogen deficiency, as observed by Ravazzolo et al. (2019). *ASN3* and *ASN4* transcripts were more abundant in the third leaf and stem of WT, suggesting that SLs could be involved in the asparagine accumulation observed under various abiotic and biotic stresses in different plant species (Lea et al. 2007). Furthermore, *LBD7* and *ROPGEF* emerged as potential molecular targets involved in SL‐mediated regulation of drought stress responses in the stem, while *NAC1* appeared to play a similar role in the leaves.

To obtain a more comprehensive understanding of the molecular events occurring in roots of the two genotypes, and therefore attributable to SLs, under different stress conditions, we employed an untargeted RNA‐seq‐based approach.

Hereafter, we will discuss only the pathways enriched in the comparison between the two genotypes in response to the stresses, and not those enriched in the comparison between control and stress conditions within each individual genotype.

Although water stress most severely affected seedling performance and gene expression, nitrogen starvation caused the greatest transcriptional divergence between genotypes, as shown by PCA (Figure 6A), heatmap (Figure 6B), and Venn diagram analyses (Figure 6C).

The only enriched pathway displaying a unidirectional response to WS was *starch and sucrose metabolism* (Figure 7C). Although the number of genes involved was relatively small, they were all more highly expressed in the WT than in the mutant (Table 1A). As reported in previous studies, during drought stress, plants modulate carbohydrate metabolism to promote the accumulation of soluble sugars, which stabilize cellular membranes and act as osmoprotectants (Mohammadkhani and Heidari 2008; Sami et al. 2016). These findings suggest that SLs may contribute to the regulation of this metabolic pathway, thereby enhancing drought tolerance.

Otherwise, under 0N, the number of significantly enriched pathways was markedly higher and mostly related to C and N metabolism, with most genes showing lower expression in *zmccd8* relative to the WT.

Many transcripts belonging to the *phenylpropanoid biosynthesis* pathway were identified (Figure 7B). This metabolic process provides precursors for flavonoids, key antioxidant molecules involved in scavenging reactive oxygen species (ROS) generated during abiotic stress (Shomali et al. 2022). These findings are consistent with the above data showing higher content of anthocyanins, a subclass of flavonoids (Kumar et al. 2023) in WT leaves under 0N and suggest that the mutant, impaired in SL production, has a reduced capacity to cope with oxidative stress, which may limit its physiological plasticity and adaptability to N deprivation. The involvement of the control of ROS homeostasis in the modulation of the maize root response to nitrogen has already been hypothesized by Trevisan et al. (2019).

Furthermore, we observed that the transcription of a group of genes belonging to the *MAPK signaling pathway*, associated with MAPK, ABA, ROS, and Ca^2+^, and known to be interconnected (Korek and Marzec 2023) as well as to play key roles in plant stress responses (Ravi et al. 2023; Rogers and Munné‐Bosch 2016), were significantly less expressed in *zmccd8* roots under nitrogen deficiency. SLs may influence MAPK cascades by modulating ABA sensitivity and ROS homeostasis, ultimately impacting stress‐related signaling pathways. While direct evidence for SL‐mediated regulation of MAPK modules in cereals remains limited, recent studies in horticultural species have shown that treatment with the synthetic SL analogue GR24 induces MAPK‐related response (Zhang et al. 2020; Zhang, Wei, et al. 2022; Zhang, Zhang, et al. 2022), suggesting that similar mechanisms may also operate in cereals, although they remain to be fully elucidated. In addition, within the *biosynthesis of plant secondary metabolites* pathway, several genes associated with the benzoxazinoid (BXD) biosynthesis were downregulated in the roots of the *zmccd8* mutant (Table S5). Previous studies suggest that BXDs, beside playing a key role in pathogen defense in grasses (Niemeyer 2009), may also contribute to iron chelation, thereby enhancing its acquisition in maize (Hu et al. 2018; Zhou et al. 2018). Within the same functional category, several DEGs were associated with the nicotianamine synthase and mugineic acid biosynthesis pathways (Table S4), both fundamental for Fe homeostasis in maize (Ma and Nomoto 1996; Li et al. 2018). These findings are consistent with previous results (Quaggiotti et al. 2024) and confirm a role for SLs in iron metabolism. In addition, BXD production occurs at epidermal rupture sites during crown root emergence to protect from pathogens in both barley (Nguyen et al. 2024) and maize (Robert et al. 2012). The impaired SL production seems to affect both BXD biosynthesis and crown root development, as observed in *zmccd8*. These results not only reinforce the idea that SLs are crucial for the development of adventitious roots in response to nitrogen deficiency but also contribute to a better understanding of the biochemical mechanisms associated with this process.

As far as *nitrogen metabolism* pathway is concerned, a decreased expression of two genes encoding nitrate reductase and one nitrite reductase (Table S5), together with that of several genes involved in nitrate and ammonium transport (Table 1B), was observed in *zmccd8*, indicating a likely lower nitrogen assimilation capacity.

The central role of SLs in the response to nitrogen is further supported by the high number of genes encoding transcription factors showing higher expression in WT than in the mutant under 0N (Table S6). Under WS, the number of differentially expressed TFs between the two genotypes was markedly lower (36). Previous omics studies have shown that the TF families most responsive to nitrogen deficiency in maize roots include MYB, NAC, WRKY, ERF, and bHLH (Wang et al. 2022; Fang et al. 2024). In our dataset, these same families also displayed the largest expression differences between the two genotypes under 0N. Notably, several TFs that showed lower expression in the mutant than in the WT, specifically *ZmMYB32, ZmMYB121, ZmMYB192, ZmNAC4, ZmbHLH91, ZmCA3P9, ZmHB22*, and *ZmRAV1*, were also reported to be induced by nitrogen deficiency in maize roots in a previous study (Ma et al. 2020). This observation suggests that these transcription factors represent key regulatory components of the SL‐mediated responses to nitrogen stress.

Finally, the identification of a high number of DEGs encoding ethylene‐responsive transcription factors (ERFs) (Table S6) and further components of the ethylene biosynthesis and signaling (Figure 8) suggests that SL‐mediated regulation of maize root responses to nitrogen deficiency may also involve this hormone, and that this interplay could be specifically associated with the regulation of adventitious roots development observed in response to N starvation, and lacking in the *zmccd8*. Indeed, in barley, the emergence of crown roots is associated with programmed cell death processes mediated by ET and ROS (Nguyen et al. 2024) and the number of crown roots was significantly reduced in rice seedlings treated with 1‐MCP, an ET signaling inhibitor (Singh et al. 2022). It is also worth noting that leaf senescence induced by nitrogen deficiency is known to be mediated by ET signaling (Khan et al. 2015; Xing et al. 2024). In this context, the present results seem to suggest that the SL‐dependent nutrient remobilization observed in leaves during nitrogen starvation (Figures 3, 4) might also depend on the cooperation between SLs and ET. Further studies will be required to validate and better elucidate this hypothesis.

In conclusion, our results demonstrate that SLs are involved in the maize responses to all the stresses examined, but their role is particularly critical under N deficiency. This condition most clearly revealed pronounced physiological and transcriptional differences between WT and the SL‐deficient *zmccd8* mutant. The absence of stress‐induced leaf senescence in the mutant, together with impaired nutrient remobilization, reduced adventitious root formation, lower expression of nitrogen transporters and assimilation genes, and attenuated activation of stress‐related TFs, indicates that SLs are central regulators of the adaptive response to low nitrogen availability.

Although water stress exerted the strongest overall impact on plant performance, the largely similar responses of the two genotypes suggest that SLs contribute only partially to drought adaptation under the conditions tested. In contrast, nitrogen deprivation uncovered a predominant and coordinated role of SLs in modulating root system architecture, secondary metabolism, redox and MAPK‐related signaling, and the expression of key regulatory TFs.

Collectively, these results position strigolactones as pivotal components of the hormonal network governing nitrogen sensing, nutrient remobilization, and root plasticity in maize, possibly acting in concert with ET. This work provides a framework for understanding SL‐mediated regulation of NUE and supports future strategies aimed at enhancing crop resilience under nutrient‐limiting and climate change–related stress conditions.

## Author Contributions

L.B., L.R. and S.Q. conceptualized and designed the research; S.Q. obtained funds to support the project; L.B., C.C. and S.Q. performed the experiments; L.B. and S.Q. analyzed the data and wrote the manuscript; R.H. and M.W. provided valuable input and critically reviewed the manuscript during its preparation. All authors read and approved the final version.

## Funding

This work was supported by The University of Padova (DOR: 2023–2024). L.B. was supported by a PhD fellowship PNRR DM 118/2023. L.R. was supported by a grant from Pipeline‐Unimpresa 2020.

## Disclosure

None was used for text or figures.

## Supporting information


**Figure S1:** Analysis of leaf number and adventitious roots percentage in WT and *zmccd8* maize plants under different growth conditions.
**Figure S2:** Gene Ontology (GO) enrichment analysis showing the top 30 terms under control, nitrogen deficiency, and water stress conditions.

**Table S1:** List of genes and corresponding primers used for qRT‐PCR (Excel file).

**Table S2:** List of identified novel transcripts (Excel file).

**Table S3:** Summary of sequencing data and quality statistics (Excel file).

**Table S4:** List of differentially expressed genes between the two genotypes under control conditions (Excel file).

**Table S5:** List of enriched pathways under the three tested conditions (Excel file).

**Table S6:** List of transcription factors and regulators differentially expressed between the two genotypes under the three tested conditions (Excel file).

**Table S7:** List of enriched GO terms under the three tested conditions (Excel file).

## Data Availability

The data that support the findings of this study (raw RNA‐sequencing FASTQ files and processed FPKM matrix) are openly available in the NCBI Gene Expression Omnibus at https://www.ncbi.nlm.nih.gov/geo/, reference number GSE318738.
