# Supplementary material for: Nitrogen Deficiency Outperforms Water Stress in Triggering Strigolactone‐Dependent Responses in Maize
Source: Physiol Plant. 2026 Jul 9;178(4):e70987. doi: 10.1111/ppl.70987 (PMC13348872; doi:10.1111/ppl.70987)
Supplement: Supplementary file 1 — Figure S1: Analysis of leaf number and adventitious roots percentage in WT and zmccd8 maize plants under different growth conditions. Figure S2: Gene Ontology (GO) enrichment analysis showing the top 30 terms under control, nitrogen deficiency, and water stress conditions. [file PPL-178-e70987-s008.pdf]

Nitrogen Deficiency Outperforms Water Stress in Triggering Strigolactone-Dependent Responses in Maize

Leonardo Buzzicotti [1], Claudia Camilletti [1], Laura Ravazzolo [1], Markus Wirtz [2], Rüdiger Hell [2] and Silvia Quaggiotti [1,*]

[1] Department of Agronomy, Food, Natural Resources, Animals and Environment, University of Padova, Viale Dell' università 16, 35020 Legnaro, Italy

[2] Centre for Organismal Studies (COS), Heidelberg University, Im Neuenheimer Feld 230, 69120 Heidelberg, Germany

[*] Corresponding author: silvia.quaggiotti@unipd.it

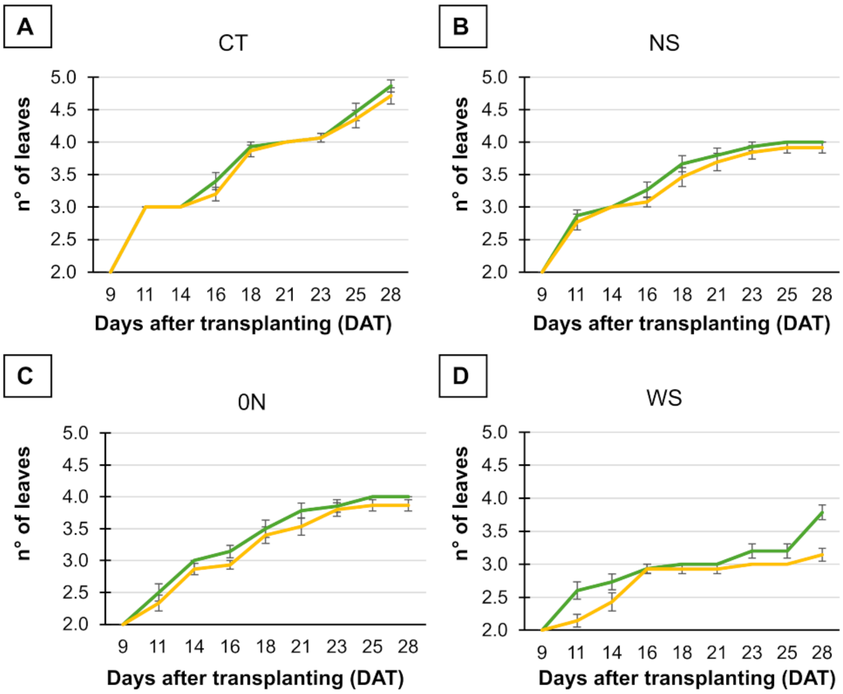

E

		DAT								
		9	11	14	16	18	21	23	25	28
**CT**	**WT**	ns	a	a	a	a	a	a	a	a
	zmccd8	ns	a	a	ab	ab	a	a	ab	a
**NS**	**WT**	ns	ab	a	ab	abc	ab	a	bc	b
	zmccd8	ns	abc	a	ab	bcd	ab	a	c	b
**0N**	**WT**	ns	bcd	a	ab	abc	ab	a	bc	b
	zmccd8	ns	cd	a	b	cd	b	a	c	b
**WS**	**WT**	ns	abc	ab	b	de	c	b	d	b
	zmccd8	ns	d	b	b	e	c	b	d	c

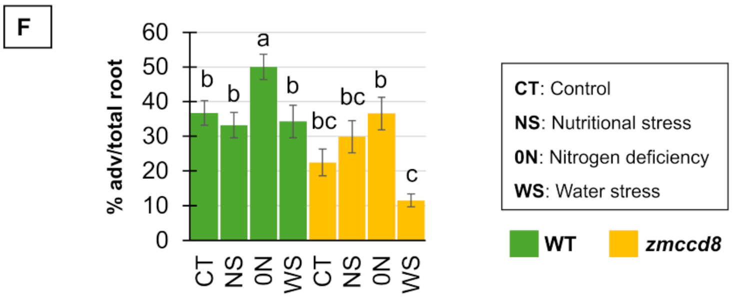

Figure S1. Number of leaves of WT (green) and *zmccd8* (yellow) maize plants during the last 20 days of growth in vermiculite under different conditions: (A) control , (B) complete nutrient deprivation , (C) nitrogen deficiency , and (D) water stress. Error bars represent mean ± SE (n = 15). Statistical analysis was performed using one-way ANOVA followed by Tukey's HSD test (E). Different letters indicate statistically significant differences ($P < 0.05$). Percentage of adventitious roots relative to total root weight 28 days after transplanting (DAT) (F).

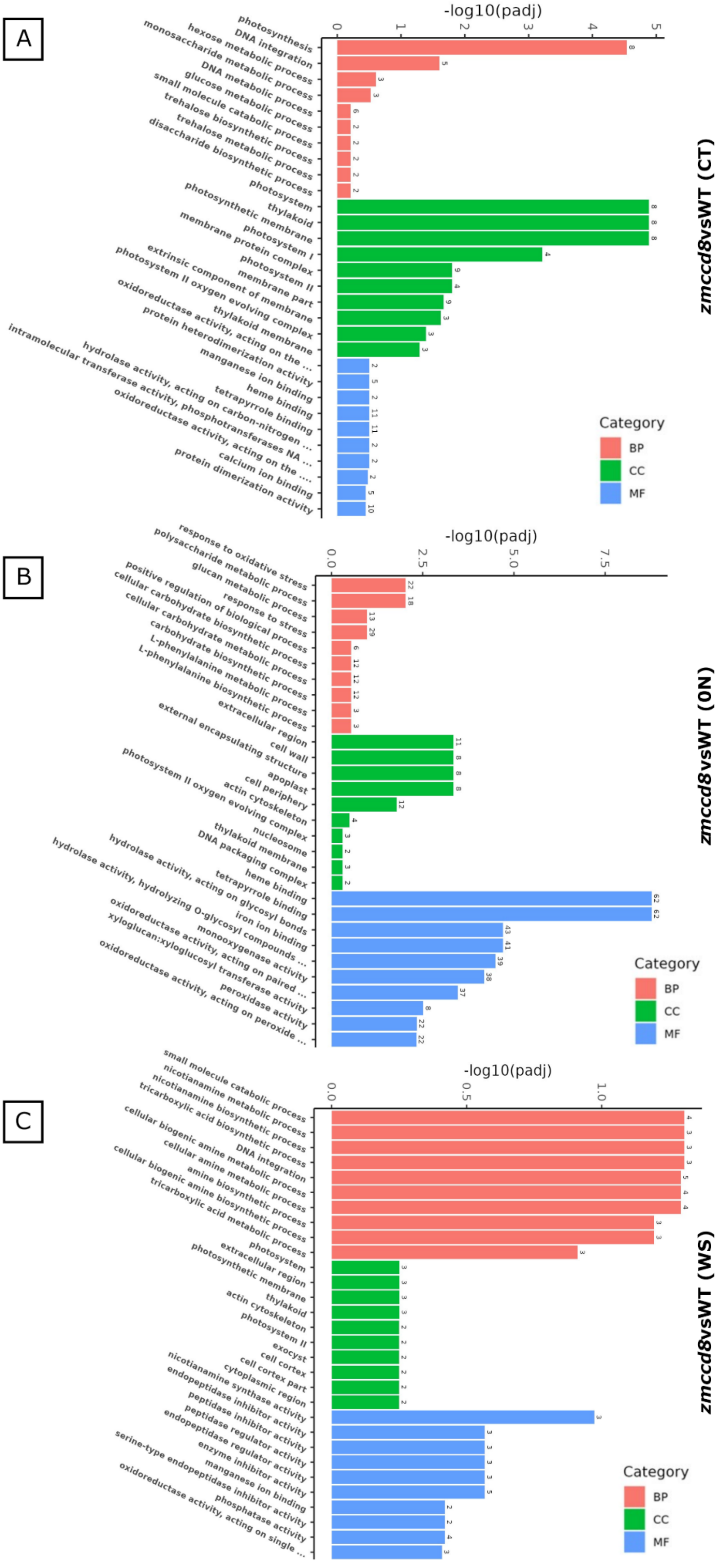

Figure S2. Top 30 enriched GO terms and their significance levels, expressed as $-\log_{10}$(padj), under the three experimental conditions: control (A), nitrogen deficiency (B), and water stress (C). Different colors represent distinct functional categories: biological process (red), cellular component (green), and molecular function (blue).
